# Exploring the Contribution of Proprioceptive Reflexes to Balance Control in Perturbed Standing

**DOI:** 10.3389/fbioe.2020.00866

**Published:** 2020-08-28

**Authors:** Anne D. Koelewijn, Auke J. Ijspeert

**Affiliations:** ^1^Biorobotics Laboratory, Institute of Bioengineering, École Polytechnique Fédérale de Lausanne, Lausanne, Switzerland; ^2^Machine Learning and Data Analytics Lab, Faculty of Engineering, Friedrich-Alexander-Universität Erlangen-Nürnberg, Erlangen, Germany

**Keywords:** balance control, reflexes, perturbed standing, neuromusculoskeletal simulation, proprioception

## Abstract

Humans control balance using different feedback loops involving the vestibular system, the visual system, and proprioception. In this article, we focus on proprioception and explore the contribution of reflexes based on force and length feedback to standing balance. In particular, we address the questions of how much proprioception alone could explain balance control, and whether one modality, force or length feedback, is more important than the other. A sagittal plane neuro-musculoskeletal model was developed with six degrees of freedom and nine muscles in each leg. A controller was designed using proprioceptive reflexes and a dead zone. No feedback control was applied inside the dead zone. Reflexes were active once the center of mass moved outside the dead zone. Controller parameters were found by solving an optimization problem, where effort was minimized while the neuro-musculoskeletal model should remain standing upright on a perturbed platform. The ground was perturbed with random square pulses in the sagittal plane with different amplitudes and durations. The optimization was solved for three controllers: using force and length feedback (base model), using only force feedback, and using only length feedback. Simulations were compared to human data from previous work, where an experiment with the same perturbation signal was performed. The optimized controller yielded a similar posture, since average joint angles were within 5 degrees of the experimental average joint angles. The joint angles of the base model, the length only model, and the force only model correlated weakly (ankle) to moderately with the experimental joint angles. The ankle moment correlated weakly to moderately with the experimental ankle moment, while the hip and knee moment were only weakly correlated, or not at all. The time series of the joint angles showed that the length feedback model was better able to explain the experimental joint angles than the force feedback model. Changes in time delay affected the correlation of the joint angles and joint moments. The objective of effort minimization yielded lower joint moments than in the experiment, suggesting that other objectives are also important in balance control, which cause an increase in effort and thus larger joint moments.

## 1. Introduction

Balancing is a complex task, where the aim is to avoid deviations from an upright and unstable position, since these deviations could lead to falls. Despite most falls occurring during walking, balance has often been studied in standing (Winter, 1995). A control system should be studied via an indirect approach, with perturbations, to identify the sensitivity of the controller and plant to noise, because a direct approach incorrectly assumes the control and state to be independent and is only appropriate for open-loop systems (Van der Kooij et al., 2005). Balance control has been identified through perturbed standing data for different scientific, robotic, and clinical applications (Wang and van den Bogert, 2020a), e.g., to provide insight into the different sensors used to balance, or to implement the identified controller into a robot.

The human is generally modeled as an inverted pendulum to study standing balance (e.g., Van der Kooij et al., 1999; Mergner et al., 2003), since inverted pendulum motion correlates with human motion in standing (Gage et al., 2004). This approach allows for the application of classical control theories to humans (Winter, 1995). A one-link inverted pendulum can be used to study the ankle strategy (Runge et al., 1999), which is active during slower perturbations. A two-link pendulum can be used to study the hip strategy, which is used for faster perturbations (Runge et al., 1999). The dynamics are typically linearized and feedback is applied to the pendulum's state, with the underlying assumption that the central nervous system is able to recover this information based on the available sensors (Van der Kooij et al., 1999).

However, these simple inverted pendulum models might not capture all aspects of standing balance. A principal component analysis of quiet standing showed that the motion in the ankle, knee and hip are similarly important, indicating that standing balance should be studied with a pendulum with more than one segment (Pinter et al., 2008). Another study also found that when the knee was not modeled, the center of mass (COM) acceleration was greatly overestimated (Yamamoto et al., 2015). Furthermore, previous work identified event-based intermittent control in tasks similar to standing, meaning that feedback is applied only after a trigger event (Loram et al., 2011, 2012), while an inverted pendulum reduces the base of support from the foot to a single point.

Therefore, we would like to investigate standing balance with a neuro-musculoskeletal model including the knee and a foot. Such a model allows us to investigate the contribution of reflexes to balance and also investigate similarities between control of walking and standing. Common muscle synergies exist between walking and standing (Chvatal and Ting, 2013), meaning that one neural control model could potentially be applied to walking and standing. Previously, a reflex controller was shown to replicate normal human walking (Geyer and Herr, 2010; Song and Geyer, 2015), which has since been applied to control exoskeletons (Wu et al., 2017), prostheses (Eilenberg et al., 2010), and a humanoid robot (Van der Noot et al., 2015), and was extended, for example to include frontal muscles as well (Song and Geyer, 2015).

We would like to investigate to what extent a similar reflex model can replicate behaviors observed in experiments of perturbed standing. The sensor fusion of, and interaction between the different proprioceptive, vestibular, visual, and other sensors has been studied extensively (Van der Kooij et al., 1999; Mergner et al., 2003; Jeka et al., 2004; Jiang et al., 2017), but the individual contributions of different sensor systems less so. Recently, perturbed standing balance was studied using a reflex controller, which was extended with a model of the supra-spinal system. This control was shown to be representative of human balance (Suzuki and Geyer, 2018a). However, Suzuki and Geyer used time delays of up to 20 ms, while reflexes have a time delay of at least 35 ms (De Groote et al., 2017). Furthermore, the resulting motions were not directly compared to experimental motions with the same perturbations, as was done by Van der Kooij and De Vlugt (2007).

Therefore, in this paper, we aim to investigate how well a proprioceptive control system with realistic time delay can explain kinetics and kinematics of perturbed standing. We replicated a perturbed standing experiment, where a person stood on a platform that was randomly displaced in the sagittal plane. This perturbed standing experiment was recreated in simulation to be able to compare against the same disturbances that were applied in the experiment. The simulation was controlled using length and force feedback. We address the questions of how much proprioception alone could explain balance control, and whether one modality, force or length feedback, is more important than the other.

## 2. Methods

### 2.1. Musculoskeletal Model

**Figure 1:**
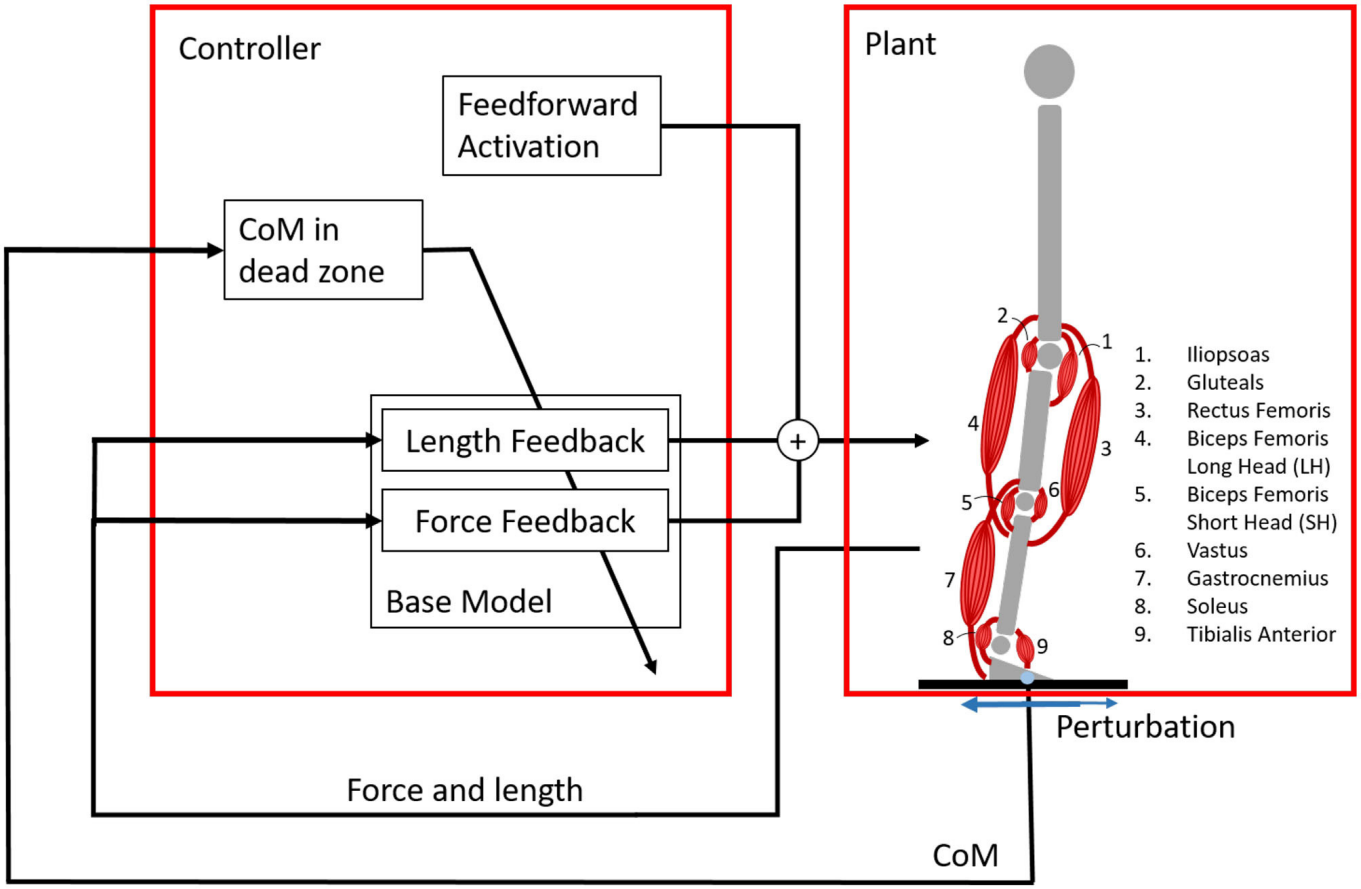
Overview of the system with the reflex controller and the plant, the musculoskeletal model. The musculoskeletal model is standing on a platform, that is perturbed in the sagittal plane by a random square wave signal. The musculoskeletal model has nine degrees of freedom, but effectively six are used because the control is the same in the left and right leg. Each leg is operated by nine muscles. Each muscle is controlled by feedforward activation and reflex loops based on force and length information. These reflex loops are only active when the center of mass (COM) is outside of a dead zone inside the base of support. Three different controllers are created: the base model with length and force feedback, a length feedback model, and a force feedback model. The control outputs nine different signals, one for each muscle in both legs.

We investigate the system shown in Figure 1. The musculoskeletal model was modeled with a floating base approach. The model has nine degrees of freedom and nine muscles in each leg. The model consists of seven segments (trunk including head, two thighs, two shanks, and two feet) connected by revolute joints. Note that the control is the same in both legs, meaning that effectively there are only six degrees of freedom: the position and orientation of the trunk, the hip angle, the knee angle, and the ankle angle.

The rigid body model was modeled in Webots (Cyberbotics Ltd., Lausanne, Switzerland) (Michel, 2004) and used previously to investigate the effect of central pattern generators (Dzeladini et al., 2014). Now, a platform was added to the environment, between the ground and the human model. This platform moved in the sagittal plane to replicate a perturbed standing experiment. The platform mass was high (1,000 kg) to prevent any inertial effects from the human model.

The model was controlled with nine muscles in each leg, which were derived from (Geyer and Herr, 2010). These muscles were four element Hill-type muscles with a contractile element, a parallel elastic element with damping, a series elastic element, and a base elastic element to avoid collapsing of the series elastic element (Geyer and Herr, 2010). We added the rectus femoris and short head of the biceps femoris such that all mono- and bi-articular muscles of the human leg were modeled. The importance of the rectus femoris on balance has also been highlighted previously (Clark, 2012). Table 1 shows the muscle parameters of these muscles, which were based on Koelewijn and Van den Bogert (2016) and Delp et al. (2007). Parameters of all muscles can be found in the Tables S1, S2. Note that the maximum isometric force in the tibialis anterior was increased to *F*_*max*_ = 4, 000 N, since preliminary work showed that the muscle was too weak to withstand the perturbations with the maximum isometric force used by Geyer and Herr (2010). This number is high, but will be scaled by the activation. The muscle dynamics and control were coded in Python 2.7.

**Table 1:** Muscle parameters of the two added muscles.

**Name**	**Rectus femoris**	**Biceps femoris short head**
Optimal contractile element length	8.1 cm	12 cm
Maximum isometric force	1,200 N	1,200 N
Series element slack length	35 cm	10 cm
Pennation	0.5	0.7

### 2.2. Control Design

Each muscle was controlled using three components: a feedforward signal, a force feedback signal, and a length feedback signal. The length feedback represented the signals from the muscle spindles, while the force feedback represented the signal from the Golgi tendon organ. Different models have been suggested for muscle spindles, but we chose the simplest model, similar to the model used by Geyer and Herr (2010), to limit the search space of the optimization. Feedback was only applied once the COM of the full body was outside the dead zone inside the base of support. The COM location was shown to be very important in balance control (Welch and Ting, 2008).

#### 2.2.1. Feedback Control

The muscle stimulation from force feedback was determined as follows:

(1) $$\begin{array}{l} u_{FFB}=G_{FFB}F_{SEE}\left(t-\Delta t\right) \end{array}$$

where *G*_*FFB*_ is the gain of the force feedback, and *F*_*SEE*_(*t* − Δ*t*) the force in the series elastic element with a delay of Δ*t*.

The muscle stimulation from length feedback was determined as follows:

(2) $$\begin{array}{l} u_{LFB}=G_{LFB}\left[l_{CE}\left(t-\Delta t\right)-l_{CE\left(off\right)}\right]\text{ if }l_{CE}\left(t-\Delta t\right)>l_{CE\left(off\right)} \end{array}$$

where *G*_*LFB*_ is the gain of the length feedback, *l*_*CE*_(*t* − Δ*t*) the time-delayed contractile element length, and *l*_*CE*(*off*)_ the offset length.

The time delays are given in Table 2. The third column provides the reference(s) that were used to define the time delay. These references measured the time between a muscle sensor stimulus and a response seen at an electromyography (EMG) sensor. No clear reference was found for the hip muscles. Carpenter et al. (1999) mention that in lateral direction, hip responses can be as quick as 25 ms, but it is unsure if responses are as fast in the sagittal plane. Therefore, a time delay of 40 ms was chosen, which is the fastest of all muscles, but not as fast as the lateral time delay.

**Table 2:** Time delays used in the controller.

**Muscle**	**Time delay [ms]**	**References**
Tibialis Anterior	35	Carpenter et al., 1999
Soleus	44	Carpenter et al., 1999
Gastrocnemius	54	Shultz et al., 2001
Vastus	100	Shultz et al., 2001; Hedayatpour and Falla, 2014
Biceps Femoris Short Head	60	Shultz et al., 2001
Biceps Femoris Long Head	60	Shultz et al., 2001
Rectus Femoris	82	Carpenter et al., 1999
Gluteals	40	-
Iliopsoas	40	-

#### 2.2.2. Dead Zone Without Feedback Control

Feedback control was only active once the model's COM moved outside the dead zone. The dead zone is an area inside the base of support. When the COM is inside this area, the model is considered to be balanced and no corrections are required. Preliminary results showed that without the dead zone, the control was very jerky and not smooth. Humans stand stably whenever their COM is inside the base of support, meaning that there is more than one stable stance configuration. The COM location inside the base of support is adapted depending on the task (Le Mouel and Brette, 2017). Therefore, the feedback control was activated only when the COM was close to or past the edges of the base of support. This means that an event-based, intermittent control was designed. Intermittent control is observed in humans in tracking tasks (Loram et al., 2012) and was suggested as a possible control approach in standing as well (Collins and De Luca, 1993; Van der Kooij and De Vlugt, 2007).

We defined the dead zone using distance and transition parameters. The distance parameters, *x*_*toe*_ and *x*_*heel*_ defined the size of the dead zone from the heel side to the toe side. The transition parameters, *z*_*heel*_ and *z*_*toe*_, smoothed the transition between no feedback and feedback to make the control less jerky. It defined the area where the feedback gain increased linearly from zero to its maximum value of *G*_*FBB*_ or *G*_*LFB*_ (see also Figure S1). The sign of the gain was dependent was reversed between the heel and toe side. The COM was extracted from Webots in real time.

(3) $$\begin{array}{l} G=\{\begin{array}{ll} -G & \text{if}:\;x_{CoM}>x_{heel}\left(1-z_{heel}\right)\\ -\left(x_{heel}-x_{CoM}\right)/\left(z_{heel}x_{heel}\right)G & \text{if}:\;x_{heel}\left(1-z_{heel}\right)\leq x_{CoM}<x_{heel}\\ 0 & \text{if}:\;x_{heel}\leq x_{CoM}\leq x_{toe}\\ \left(x_{CoM}-x_{toe}\right)/\left(z_{toe}x_{toe}\right)G & \text{if}:\;x_{toe}<x_{CoM}\leq x_{toe}\left(1+z_{toe}\right)\\ G & \text{if }x_{CoM}>x_{toe}\left(1+z_{toe}\right) \end{array} \end{array}$$

### 2.3. Optimization and Simulation Approach

Each simulation was run as follows. First, a joint angle control was applied in Webots for 1 s. This allowed the muscles to find an equilibrium position. Preliminary work showed that it was very hard to control the model if it was controlled by muscles from the start of the simulation. Then, the model would stand still using muscle-control for another second, after which a perturbation signal was applied until the full simulation lasted 110 s.

Controller parameters were found by solving an optimization problem using the perturbed simulations. The following parameters were optimized: the feedforward muscle input, the parameters of the dead zone, ${\text{z}}_{safe}={\left[x_{toe}\;x_{heel}\;z_{toe}\;z_{heel}\right]}^{T}$, the gains of the force and length feedback (Equations 1 and 2) and the length offset of the length feedback (Equation 2). The parameters in the left and right leg were equal. To reduce the size of the search space, the gains were equal and opposite between the situation when the COM was location on the heel- or the toe-side.

An optimization was solved to find the controller parameters such that the muscular effort was minimized, while several constraints were used to enforce the model to stand upright for the required time period. Effort minimization was used since this objective is also used in the central nervous system to create movements (Selinger et al., 2015), and is known to predict walking gaits (Ackermann and Van den Bogert, 2010). An effort objective also predicted responses in perturbed standing to measured EMG signals (Lockhart and Ting, 2007). All simulations were stopped if the time was exceeded or if height of the chest fell below 0.7 m. Therefore, a constraint was added that the simulation lasted at least *T* = 100 s. Secondly, a constraint was added that the height of the chest, *y*_*chest*_, was at least 1.3 m at the final time of the simulation. Thirdly, to avoid slipping, the horizontal ankle position, *x*_*ankle*_, was constrained throughout the simulation. This yielded the following optimization problem:

(4) $$\begin{array}{ll} \underset{\text{z}={\left[{\text{u}}_{0}\;{\text{G}}_{FFB}\;{\text{G}}_{LFB}\;{\text{l}}_{CE\left(off\right)}\;{\text{z}}_{deadzone}\right]}^{T}}{\text{minimize}} & f\left(\text{z}\right)=\frac{1}{T}\int_{t=0}^{T}\sum_{i=1}^{N_{mus}}a_{i}{\left(t\right)}^{2}\text{d}t \end{array}$$

(5) $$\begin{array}{ll} \text{Subject to}: & T>100\text{ s} \end{array}$$

(6) $$\begin{array}{l} y_{chest}\left(T\right)>1.3\text{ m} \end{array}$$

(7) $$\begin{array}{l} \frac{1}{T}\int_{0}^{T}x_{ankle}\left(t\right)\text{d}t<0.05\text{ m} \end{array}$$

where *u*_0_ is the feedforward input and *a*_*i*_ the activation of muscle *i*.

This problem was solved using single shooting and a particle swarm optimization (PSO). A lexicographic extension of PSO (Dzeladini et al., 2014) was used to ensure that the constraints were met. Each optimization was seeded from a good initial guess. This initial guess was found in preliminary work from a random initial guess. The population size was varied depending on the controller architecture. The open-loop input, *u*_0_ was bound between 0.001 and 1. All feedback gains were bound between −3 and 3 and the length offset *l*_*CE*(*off*)_ was bound between 0.2 and 1.1. The dead zone parameters, **z**_*deadzone*_, were bound to be within 10 cm of the contact point at the heel and toe for *x*_*heel*_ and *x*_*toe*_, respectively. The transition parameter was bound between 0 and 1.

The perturbation signal was taken from Wang and van den Bogert (2020a), where a 5 min perturbation signal was applied to human participants. This signal was designed using random square pulses with different amplitudes, [−5, −2.5, 0, 2.5, 5] cm, and different pulse durations, [0.25, 0.5, 0.75, 1.0, 1.25, 1.5] s (Wang and van den Bogert, 2020a). However, the optimization platform limited the simulations to be around 100 s due to technical limitations, so only part of this signal was used. The platform that was modeled in Webots was controlled to move exactly as the platform motion recorded in the experiment (Wang and van den Bogert, 2020a). The simulation modeled the perturbation signal starting at a randomly chosen start time, 53.73 s.

Three different control architectures were designed with monosynaptic feedback pathways. The base model had both force and length feedback. The length feedback model only used length feedback, while the force feedback model only used force feedback.

### 2.4. Comparison to Human Experimental Data

The resulting joint angles and joint moments were compared to experimental data provided by Wang and van den Bogert (2020b), where the same perturbation signal was applied in an experiment. One participant of this study was selected with the height and weight most similar to the height and weight of the musculoskeletal model. Ground reaction force and marker data were filtered with a second order Butterworth filter with a cut-off frequency of 16 Hz. Ground reaction force data was inertially compensated to account for belt acceleration (Hnat and Van den Bogert, 2014). Joint angles and joint moments were determined using marker orientation and a link-segment model, as described by Koelewijn et al. (2019).

The experimental data was resampled to match the sampling rate and time points of the simulation, after which the correlation between the simulation and experiment was determined using Pearson's linear correlation coefficient. The correlation coefficient and the *p*-value of the hypothesis that there is no correlation between the data were determined in MATLAB (Mathworks, Natick, MA, USA). The correlation was determined for the data used in the optimization, and repeated for a second random 100 s signal sample from the experimental data.

## 3. Results

### 3.1. Controller Parameters

Table 3 shows the optimized controller parameters for each of the controllers. The parameters *x*_*heel*_ and *x*_*toe*_ define the distance between the edge of the dead zone and the contact point at the heel and toe, respectively. Figure S1 shows how the gain varied depending on the COM position. The open-loop inputs are very similar between the different solutions, with a maximum difference of 0.12 in the soleus and gastrocnemius. Furthermore, the feedback gains are generally highest for the monoarticular hip muscles, and the ankle muscles. In the force feedback model, all reflex gains are positive, except for the tibialis anterior, while in the length feedback model positive and negative gains were found.

**Table 3:** Optimized controller parameters for each of the controllers that were found.

**Parameters**	**Muscle**	**Base**	**Length feedback**	**Force feedback**
*z*_*heel*_		0.15	0.27	0.32
*z*_*toe*_		0.71	0.67	0.53
*x*_*heel*_		5.74 cm	6.60 cm	5.99 cm
*x*_*toe*_		1.24 cm	2.66 cm	1.82 cm
*u*_0_	Iliopsoas	0.25	0.20	0.21
	Gluteals	0.14	0.034	0.14
	Rectus Femoris	0.093	0.20	0.17
	Biceps Femoris LH	0.26	0.32	0.30
	Biceps Femoris SH	0.11	0.16	0.12
	Vastus	0.26	0.24	0.30
	Gastrocnemius	0.13	0.067	0.17
	Soleus	0.26	0.33	0.21
	Tibialis Anterior	0.062	0.0076	0.045
*G*_*FFB*_	Iliopsoas	1.01		0.31
	Gluteals	0.89		1.38
	Rectus Femoris	0.24		0.50
	Biceps Femoris LH	0.53		0.11
	Biceps Femoris SH	0.25		0.48
	Vastus	−0.23		0.031
	Gastrocnemius	0.27		0.31
	Soleus	1.50		1.36
	Tibialis Anterior	−1.11		−1.45
*G*_*LFB*_	Iliopsoas	0.72	0.23	
	Gluteals	0.029	−0.45	
	Rectus Femoris	−0.54	0.12	
	Biceps Femoris LH	−1.07	−0.28	
	Biceps Femoris SH	0.66	0.22	
	Vastus	0.21	−0.042	
	Gastrocnemius	−0.23	−0.63	
	Soleus	0.29	1.15	
	Tibialis Anterior	−0.19	−0.38	
*l*_*CE*(*off*)_	Iliopsoas	1.02	0.52	
	Gluteals	0.94	0.73	
	Rectus Femoris	0.64	0.67	
	Biceps Femoris LH	0.67	0.86	
	Biceps Femoris SH	0.76	0.26	
	Vastus	0.39	0.58	
	Gastrocnemius	0.86	0.86	
	Soleus	0.98	0.50	
	Tibialis Anterior	1.09	0.70	

### 3.2. Correlations Between Experiment and Simulation

**Figure 2:**
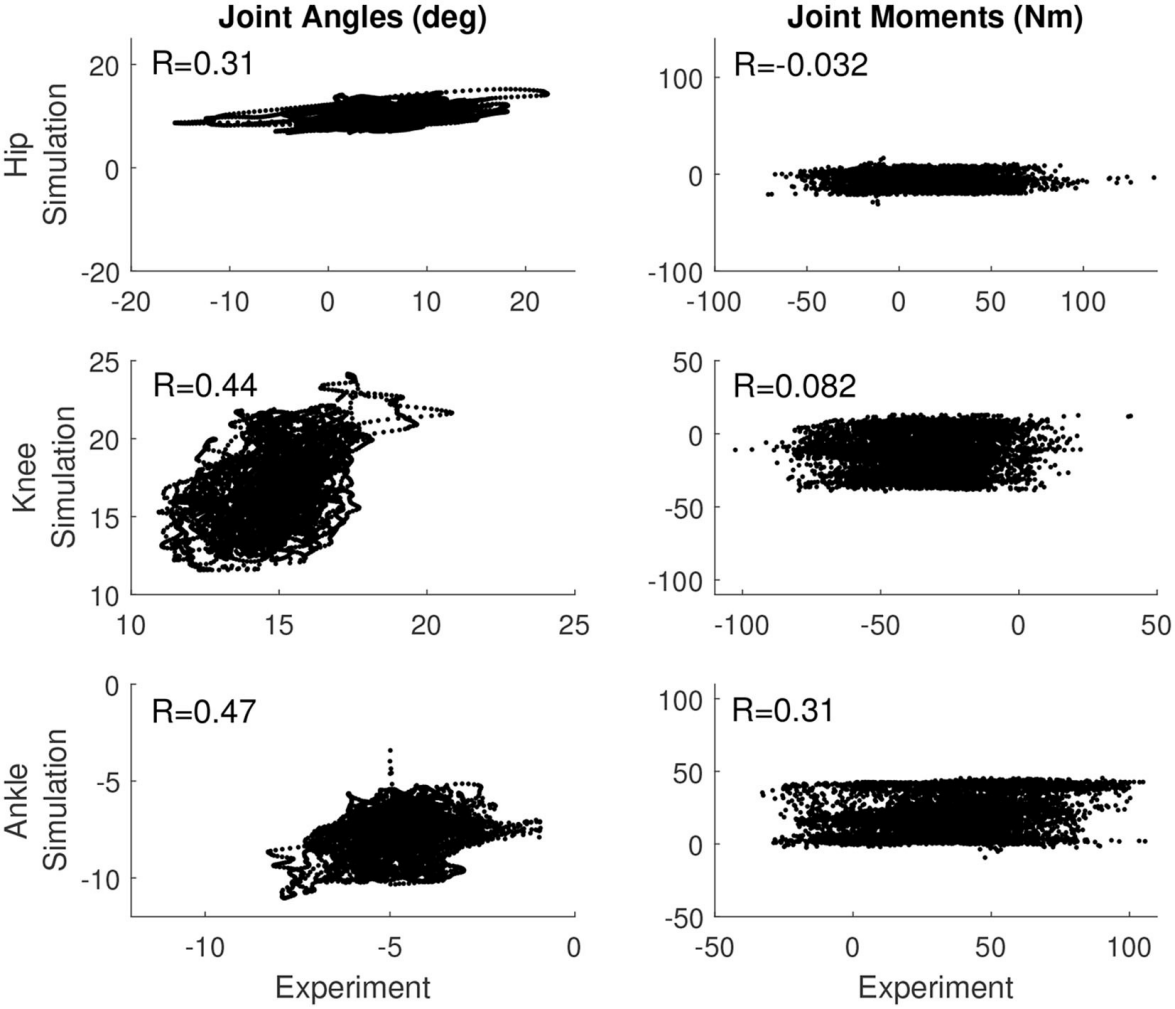
Correlation between the simulation controlled with length and force feedback and the experiment for the joint angles **(Left)** and joint moments **(Right)** for all three joints.

Table 4 reports the correlation between the simulated and experimental joint angles and joint moments for each of the control models that was used. For the base model, the hip and knee angle correlated moderately between experiment and simulation, while the ankle angle, ankle moment, and knee moment correlated weakly (see also Figure 2). The correlation in the hip moment was barely significant when correcting for multiple comparisons (*p* = 0.001). Similarly, for the length feedback model, there was a moderate correlation for the hip and knee joint angle, and a weak correlation for the ankle angle, ankle moment, and knee moment, while there was a weak negative correlation for the hip moment (*p* = 0.0001). For the force feedback model, the knee and hip joint angle correlated moderately between the simulation and experiment, while the ankle correlated weakly. Again, the coefficients of all joint angles were significant with *p* ≤ 0.0001. The simulated ankle (positive) and hip moment (negative) correlated weakly with the experiment, with a significant coefficient, while the correlation for the knee was not significant (*p* = 0.91). The correlations are also shown in Figures S2, S3.

**Table 4:** Correlation between simulated and experimental joint angles and joint moments for the experimental data used in the optimization.

**Control model**	**Joint angles**			**Joint moments**		
	**Hip**	**Knee**	**Ankle**	**Hip**	**Knee**	**Ankle**
Base model	0.31^*^	0.44^*^	0.26^*^	−0.032	0.082^*^	0.31^*^
Length feedback model	0.47^*^	0.33^*^	0.13^*^	−0.040^*^	0.10^*^	0.42^*^
Force feedback model	0.41^*^	0.47^*^	0.26^*^	−0.11^*^	−0.0011	0.25^*^

*A star indicates that the correlation is significant (p ≤ 0.0001)*.

Table 5 reports the correlation between a second, randomly chosen, 100 s sample of the experimental data. The starting times of these samples were 17.57 s for the base model, 97.20 s for the length feedback model, and 44.55 for the force feedback model. The correlations are similar to the correlations shown in Table 4, where the analysis was done with the sample used in the optimization. The correlations are lower for the joint angles, but higher for the ankle in the base model and the hip in the force feedback model. The correlations are higher for the joint moments, except the hip in the force feedback model (lower) and the knee in the length feedback model (the same).

**Table 5:** Correlation between simulated and experimental joint angles and joint moments for a random sample of the experimental data.

**Control model**	**Joint angles**			**Joint moments**		
	**Hip**	**Knee**	**Ankle**	**Hip**	**Knee**	**Ankle**
Base model	0.29^*^	0.29^*^	0.084^*^	−0.062	0.064^*^	0.38^*^
Length feedback model	0.36^*^	0.24^*^	−0.061^*^	−0.084^*^	0.10^*^	0.48^*^
Force feedback model	0.50^*^	0.44^*^	0.20^*^	−0.063^*^	−0.064	0.31^*^

*A star indicates that the correlation is significant (p ≤ 0.00001)*.

#### 3.2.1. Base Model

**Figure 3:**
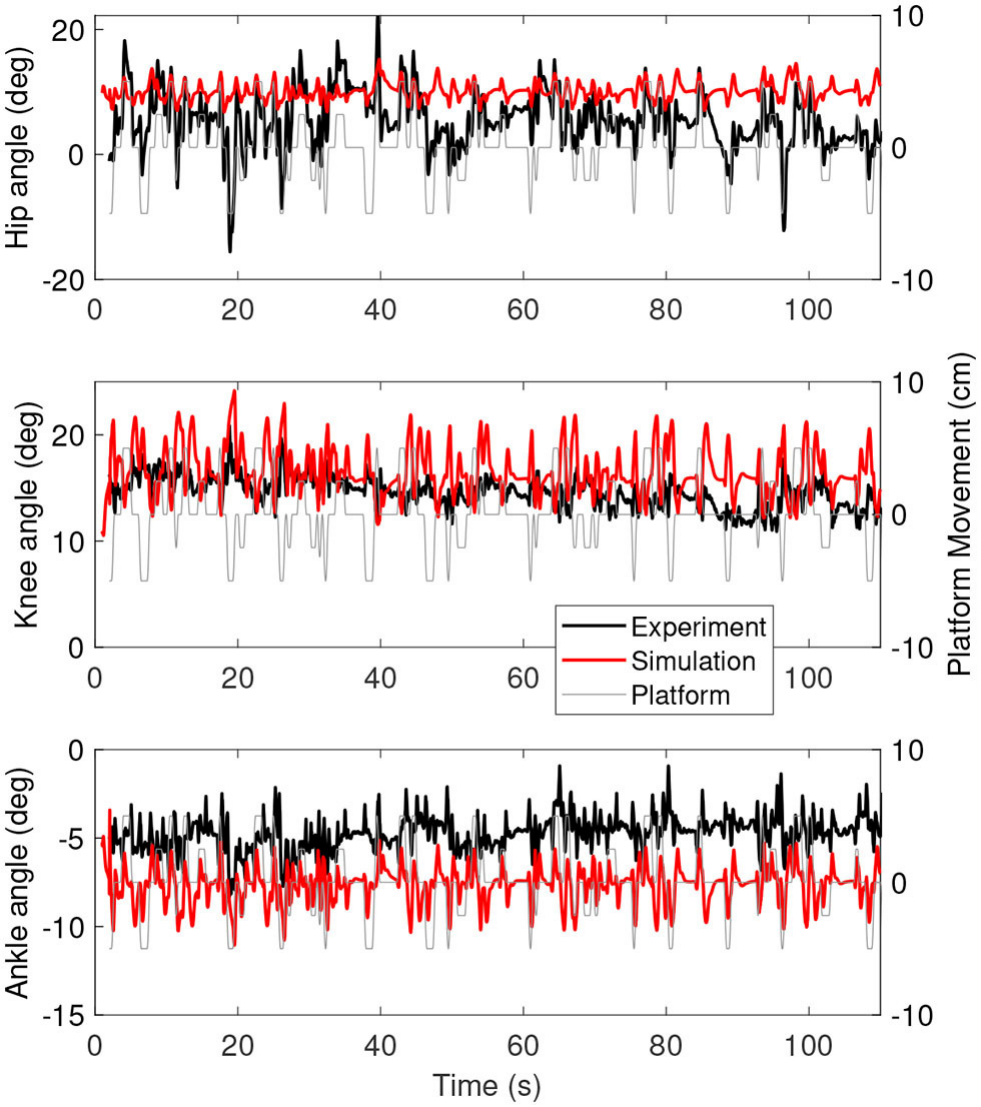
Joint angles as a function of time for the simulation controlled with length and force feedback (red) and experiment (black). The platform motion is shown in gray for reference.

**Figure 4:**
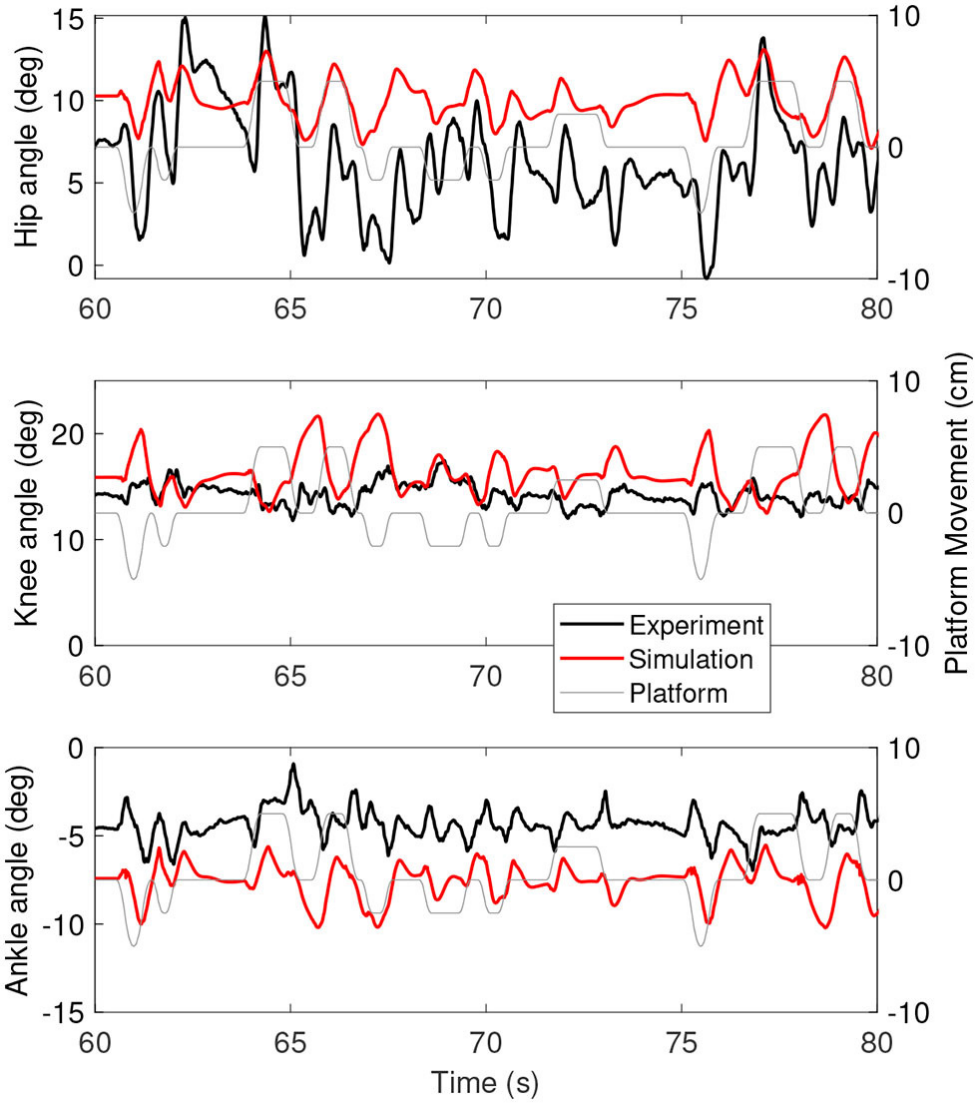
A zoom-in on the joint angles between 60 and 80 s for the simulation controlled with the base model (red) and experiment (black). The platform motion is shown in gray for reference.

Figure 3 shows the joint angles as a function of time for the simulation (red) and the experiment (black). The platform motion is also plotted for reference. The average joint angle of all joints is similar between the experiment and the simulation. The largest difference was five degrees for the hip, while the difference was one degree for the knee and three degrees for the ankle. Figure 4 shows the joint angles only for 60–80 s, where it is clear that the responses look similar between the experiment and simulation. In the knee, the simulated responses generally have a larger amplitude than the experiment, while the opposite is true for the hip and the amplitudes seem similar in the ankle.

**Figure 5:**
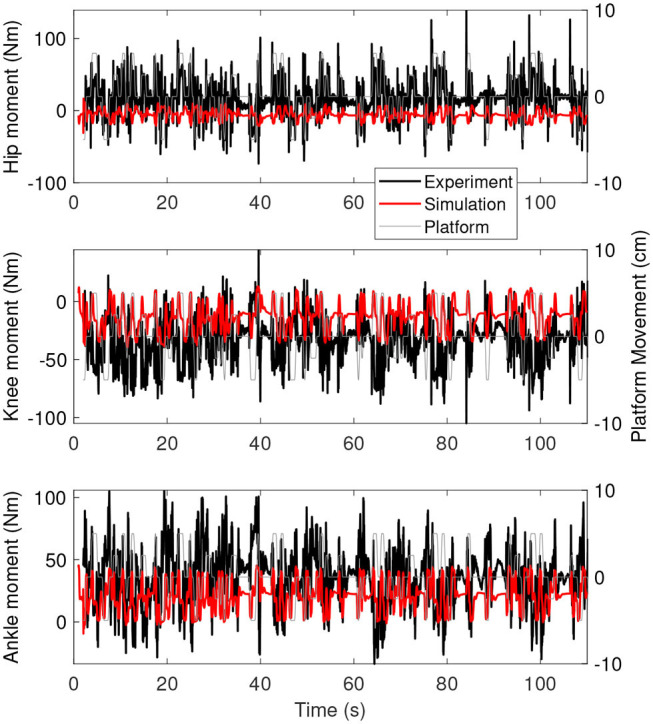
Joint moments as a function of time for the simulation controlled with length and force feedback (red) and experiment (black). The platform motion is shown in gray for reference.

Figure 5 shows the joint moments as a function of time for the simulation controlled with length and force feedback (red) and the experiment (black). The range of moments is smaller for the simulation than for the experiment, with smaller extremes in positive and negative direction.

#### 3.2.2. Single Feedback Models

**Figure 6:**
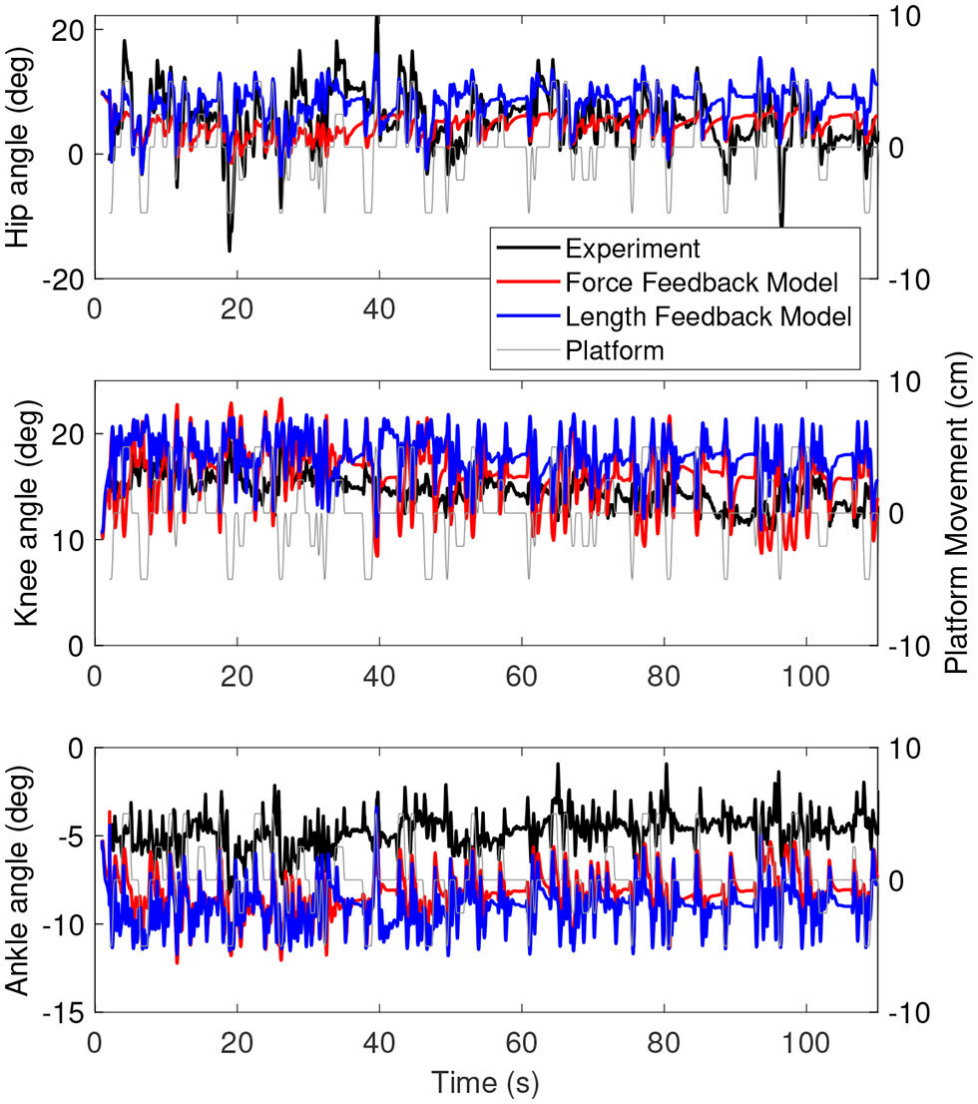
Joint angles as a function of time for the simulation controlled with the force feedback model (red), length feedback model (blue), and experiment (black). The platform motion is shown in gray for reference.

**Figure 7:**
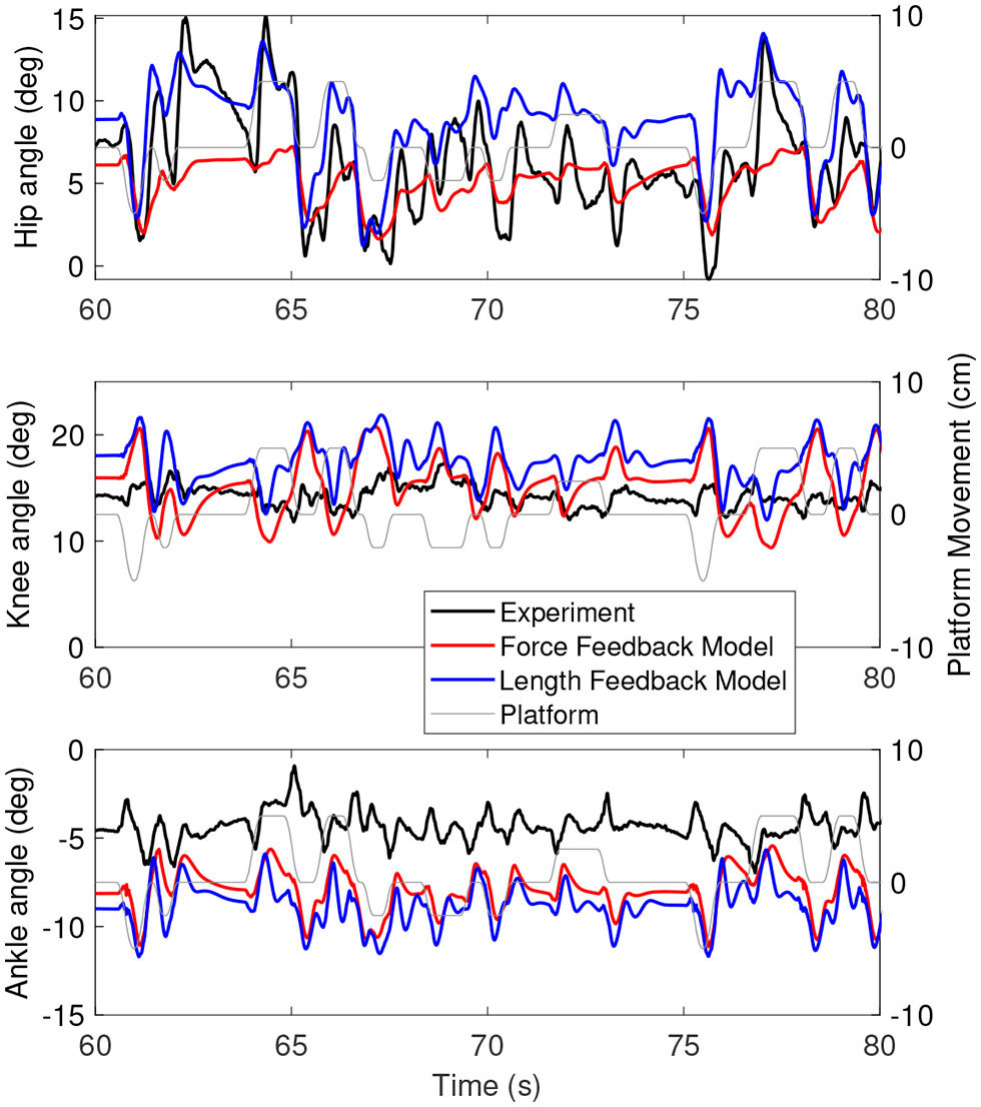
A zoom-in on the joint angles between 60 and 80 s for the simulation controlled with the force feedback model (red), length feedback model (blue), and experiment (black). The platform motion is shown in gray for reference.

Figure 6 shows the joint angles of the simulation with the force feedback model (red), the length feedback model (blue) and the experiment (black) as a function of time. The average joint angle is very similar for the length and force feedback model, and also similar to the experiment for both. The largest difference is about 3 degrees for the ankle. Figure 7 shows the same results, zoomed in on 60–80 s. This shows that the responses of the length feedback model are more similar to the experimental responses than the force feedback model. The force feedback models shows larger periods of inactivity, whereas there are responses visible in both the experiment and the length feedback model.

**Figure 8:**
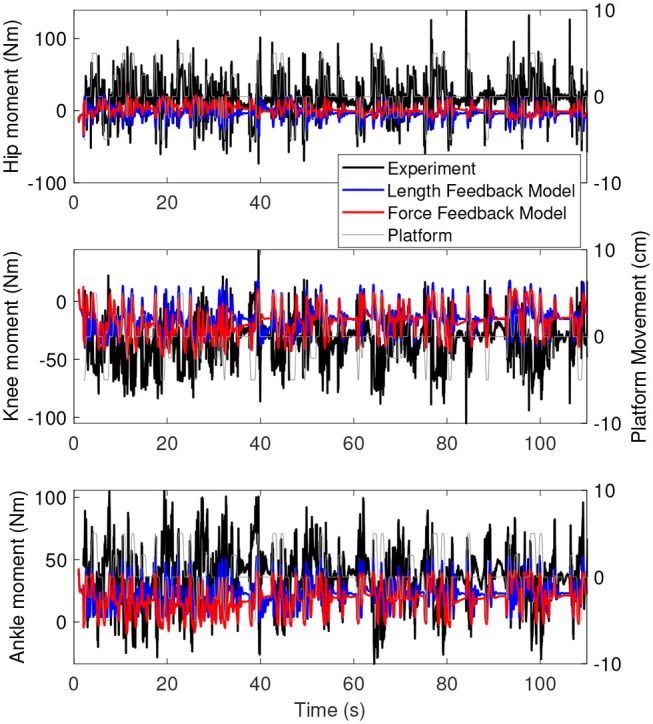
Joint moments as a function of time for the simulation controlled with length feedback (red) and experiment (black). The platform motion is shown in gray for reference.

Figure 8 shows the joint moments of the simulation with the force feedback model (red), the length feedback model (blue), and the experiment (black) as a function of time. These results are very similar to the results of the base model. The range of the moments is again smaller for the simulation than for the experiment. Similar to the joint angles, the joint moments are constant for longer periods in time for the force feedback model, while for the length feedback model, there seem to be some damped oscillations (e.g., in the knee and ankle between 80 and 90 s).

## 4. Discussion

We aimed to understand the contribution of the proprioceptive system to behavior during perturbed standing. A reflex model with both force and length reflex showed significant moderate correlation for the joint angles in all joints, and weak correlations for the knee and ankle moments. This suggests that a controller with proprioceptive reflexes can explain perturbed standing behavior. Furthermore, controllers with only force or length feedback also correlated weakly to moderately with the experiment. Analysis of the time series of the joint angles showed that the length feedback model was better able to replicate the reactions observed in the experiment than the force feedback model.

We modeled muscle spindles as pure length feedback loops to limit the search space of the optimization, while evidence exist that the spindle activity depends on both the length and velocity of the contractile element (Hasan, 1983). Recently, experiments and simulations even showed that the spindle activity is more related to force than length and velocity (Blum et al., 2017; Falisse et al., 2018). Falisse et al. (2018) showed that reflex activity during gait and passive stretches in patients with cerebral palsy was better modeled based on the contractile element force than the contractile element length. We compared contractile element length feedback to tendon force feedback, and showed the opposite in perturbed standing, since the length feedback better explained the experimental joint angles than the tendon force feedback. We expect the correlation to increase further if velocity was included into the muscle spindle model.

An important parameter in the human control system is the time delay between the sensors and the resulting corrections. We chose the time delays as much as possible according to literature on time delays (Carpenter et al., 1999; Shultz et al., 2001; Hedayatpour and Falla, 2014). We repeated the optimizations with different time delays (see Supplementary File). Again, correlations were found between the joint angles and joint moments. However, the numbers could increase or decrease up to 0.2. This indicates that the time delay of the muscles could possibly be found via a data-tracking optimization, to find the time delay where the responses of the simulation occur at the same time as the responses of the experiment.

We identified balance control by minimizing effort, whereas balance control is usually identified by minimizing the error between simulated and experimental data (e.g., Van der Kooij et al., 2005). Humans minimize some objective when walking (Bertram and Ruina, 2001), which is related to energy or effort (Ackermann and Van den Bogert, 2010; Dzeladini et al., 2014; Falisse et al., 2019). Furthermore, this energy-related objective is used by the central nervous system to continuously optimize gait patterns (Selinger et al., 2015). An effort-related objective also predicted responses to balance perturbation during standing similar to EMG signals (Lockhart and Ting, 2007). The correlation of the joint angles and moments between the simulation and experiment further indicates that this objective is important in standing balance as well. However, the resulting joint moments (Figures 5, 8) were smaller than in the experiment. This indicates that the optimal solution of the simulation might have been more energy efficient than the human behavior in the experiment. Future studies could identify additional objectives used internally by humans in perturbed standing.

Our control approach was based not only on proprioceptive reflexes, but also on the COM location. The dependency on the COM allowed for an implementation of intermittent control, which has been shown to be the approach that is used in human control (Loram et al., 2012). Previous work has shown that the COM is important for control of standing balance (Welch and Ting, 2008). However, the exact architecture of the intermittent control has not been investigated so far. We used a real time measurement of the COM, which is unrealistic, since the human body requires around 100 ms to estimate the COM position (Peterka, 2002). However, optimizing controller parameters for the base model with a 100 ms time delay on the COM position was not successful. The model was not able to withstand the full perturbation signal. Therefore, the intermittent control threshold is likely not defined by the COM. Instead, it could be defined by pressure distribution in the feet. However, we could not use the ground reaction force measurements due to measurement noise in Webots. Therefore, we used the COM as a proxy and optimized controller parameters of the base model with a 20 ms time delay on the COM position, to represent the time delay normally present between the cutaneous sensors and the spinal cord (Jenner and Stephens, 1982). The resulting control parameters, including those for the dead zone, were similar, though the length feedback gains were higher except for the biceps femoris (see Supplementary Files). Correlations were also similar, the knee and ankle angle correlated weaker (0.37 and 0.17 vs. 0.44 and 0.26 in Table 4, respectively), while the knee and ankle moment correlated stronger (0.20 and 0.41 vs. 0.082 and 0.31 in Table 4, respectively). These result suggest that the intermittent control threshold is more likely based on the foot pressure than the COM.

However, the intermittent control might have caused some differences in the joint angles between the simulations and the experiment. The joint angles (Figures 3, 6) of the simulations showed less variability when the platform was not moving than the joint angles in the experiment (e.g., around 70 s in Figure 6). This indicates that there are some corrections happening during this time, while in the simulation the COM was inside the dead zone and therefore no corrections were applied. The lack of motion when the COM was inside the dead zone is likely related to the relatively high ankle stiffness resulting from the Achilles tendon stiffness. We used the tendon stiffness from (Geyer and Herr, 2010), which is in the order of magnitude expected for the Achilles tendon (Gerus et al., 2015). However, this tendon stiffness yields an ankle stiffness that is two orders of magnitude larger than the ankle stiffness observed in quiet standing (Loram and Lakie, 2002). This observed ankle stiffness is too low to stabilize the body (Loram and Lakie, 2002), which causes random motion, whereas our much larger stiffness does stabilize the body.

We did not extend the muscle models with a short range stiffness. The muscle short range stiffness is a short term stiffness increase after a length change, faster than any neural response (De Vlugt et al., 2011). Addition of a short-range stiffness model has improved similarity between perturbed standing experiments and simulations (De Groote et al., 2017). Inspection of the time series of the joint angles showed that in general the response of the simulation was smoother and less steep than in the experiment. The short-range stiffness would allow for faster response time in the muscles, and could further improve the similarity between the simulation and experiments. We chose not to include the short-range stiffness, since our aim was to investigate the effect of the proprioceptive system. By not including this stiffness, we ensured that the observed results were due to the reflex control, and not the short-range stiffness. Future research should investigate the interaction between short-range stiffness and reflexes.

We limited the search space of the optimization by using a simple spindle model and by using an equal and opposite sign for the gain parameters if the COM was outside of the dead zone on the heel-side, compared to the toe-side. The parameter space would have more than doubled otherwise, meaning that the population size should have increased even more. With the current set-up, optimizations took about a week to run on a high performance computer with multiple clusters. Therefore, it was decided not to investigate a controller with different gains, instead of gains with an equal and opposite sign. It is likely that a better fit could be found with more controller parameters. Different numerical approaches should be investigated to allow for a larger search space, and thus more realistic models for e.g., the muscle spindles, such that our approach can also be used to investigate human control on a physiological level.

The open-loop inputs were very similar between the different controllers. The largest difference was found between the gastrocnemius and the soleus. These muscles have similar functions, meaning that there are likely multiple solutions for the open-loop input with a similar effect on the control. For the same reason, a larger variation was found in the length feedback parameters than in the force feedback parameters. The length feedback was based on two parameters, the offset and the gain. Therefore, different combinations of gains and offset could yield to similar results, while the force feedback was only based on one parameter. A different choice for the bounds of the length offset could reduce this redundancy.

The controllers were mainly evaluated on the perturbation signal used in the optimization only (100 s), while data for the complete 5 min experiment was available. We also evaluated on a second 100 s sample, with a randomly chosen start time. Correlations for both samples were similar, indicating that the evaluation using the perturbation signal from the optimization is fair. This was expected, because the optimization objective did not have a tracking term. However, the base model and force feedback model were not able to withstand the complete perturbation signal. It is expected that it is possible to find a controller for the full duration by optimizing over the full trajectory, but this was not possible due to technical limitations. The simulation time is related to the number of different perturbations that the system encounters. If this number is higher, it means that more data is available to train the controller, and the human control will be replicated more accurately. We do not expect that the conclusions of this work would be different with a larger simulation time, since we found that the reported correlations were the same for two samples of the experimental data. Future work should investigate the minimum amount of data required to find an accurate controller.

Two limitations of the current study should be mentioned. Firstly, we did not account for modulation of reflexes in different environments (Perreault et al., 2008). The controller was fitted to data of perturbed standing of a specific magnitude, and its validity in other environments with different perturbations, or quiet standing, was not tested. In quiet standing, the length of the calf muscles changes differently than expected in reflex control, since these muscles shorten during small forward motions and lengthen during backward motions (Loram et al., 2005), which is related to the fact that the ankle stiffness, as mentioned before, is lower than necessary to stabilize the body (Loram and Lakie, 2002).

Secondly, the maximum force of the tibialis anterior was relatively high, since it was not possible to find a controller with the original value used by Geyer and Herr (2010). However, the actual force is scaled by activation, which was equal to about 30% for the tibialis anterior, meaning that the maximum force in this muscle was about 1200N, which is realistic for the tibialis anterior. One consequence of the large force is that the optimization weighted the force in the tibialis anterior to a lesser extent, since a small activation already led to a large force. However, this should have only a small effect, because the tibialis anterior was the only ankle dorsiflexor muscle.

In conclusion, we investigated how well proprioceptive reflexes explain perturbed standing. A perturbed standing experiment was replicated in a simulation controlled by force and length reflexes, and only force or length reflexes. We showed a weak to moderate correlation between the joint angles and moments between the experiment and a controller optimized to minimize effort, which suggests that force and length reflexes are important for standing, but other motor control systems should be included to capture the full behavior. Correlations were similar between the length and force feedback model, but the length feedback model was slightly better able to replicate experimental motions, suggesting that length feedback is more important than force feedback in perturbed standing. Furthermore, these results were found by minimizing effort, which suggests that similar to walking, an effort-related objective is likely used in perturbed standing. The correlation was dependent on the time delay that was used, meaning that time delay should be chosen carefully in a neural control model.

## Data Availability Statement

All experimental datasets can be found in Wang and van den Bogert (2020b). The controller parameters and simulation results can be found here: doi: 10.5281/zenodo.3904097.

## Author Contributions

AK designed the study, created the controllers, performed data analysis, and drafted the article. AI conceived the work, supervised the study, acquired funding, and critically revised the article. All authors contributed to the article and approved the submitted version.

## Conflict of Interest

The authors declare that the research was conducted in the absence of any commercial or financial relationships that could be construed as a potential conflict of interest.
