# Supplementary material for: Exploring the Contribution of Proprioceptive Reflexes to Balance Control in Perturbed Standing
Source: Front Bioeng Biotechnol. 2020 Aug 28;8:866. doi: 10.3389/fbioe.2020.00866 (PMC7485384; doi:10.3389/fbioe.2020.00866)
Supplement: Supplementary file 1 [file Data_Sheet_1.PDF]

frontiers

Supplementary Material

1 MUSCLE PARAMETERS

Table S1 summarizes the muscle parameters used in this work. Table S2 summarizes the moment arm parameters that were used.

2 CONTROL GAIN FACTOR

Figure S1 shows the factor with which the gains were multiplied to implement the dead zone, using the parameters of the base model. The dead zone had a factor of 0, and at the heel the factor decreased to -1, while at the toe it increased to 1. In the neutral position, the heel control was active.

Table S1. Muscle parameters of all muscle. $l_{CE(OPT)}$ is the optimal fiber length in cm, F_{max} the maximum isometric force in N, $l_{SEE,slack}$ the slack length of the series elastic element, and $v_{CE,max}$ the maximum shortening velocity.

Name	$l_{CE(OPT)}$	F_{max}	$l_{SEE,slack}$	$v_{CE,max}$	Pennation
Gluteals	11 cm	1500 N	13 cm	12 $l_{CE(OPT)}$/s	0.5
Iliopsoas	11 cm	2000 N	10 cm	12 $l_{CE(OPT)}$/s	0.5
Rectus Femoris	8.1 cm	1200 N	35 cm	12 $l_{CE(OPT)}$/s	0.5
Biceps Femoris Long Head	10 cm	3000 N	31 cm	12 $l_{CE(OPT)}$/s	0.7
Biceps Femoris Short Head	12 cm	1200 N	10 cm	12 $l_{CE(OPT)}$/s	0.7
Vastus	8 cm	6000 N	23 cm	12 $l_{CE(OPT)}$/s	0.7
Gastrocnemius	5 cm	1500 N	40 cm	12 $l_{CE(OPT)}$/s	0.7
Soleus	4 cm	4000 N	26 cm	6 $l_{CE(OPT)}$/s	0.5
Tibialis anterior	6 cm	4000 N	24 cm	12 $l_{CE(OPT)}$/s	0.7

Table S2. Moment arm parameters of all muscles: gluteals (GLU), Iliopsoas (ILI) Rectus Femoris (RF), Biceps Femoris Long Head (BFLH), Biceps Femoris Short Head (BFSH), Vastus (VAS), Gastrocnemius (GAS), Soleus (SOL), and tibialis anterior (TA).

	Hip				Knee					Ankle		
	GLU	ILI	RF	BFLH	RF	BFLH	BFSH	VAS	GAS	GAS	SOL	TA
r_0 (cm)	10	10	8	8	4	5	5	6	5	5	5	4
ϕ_{max} (deg)	-180	-180	-180	-180	15	0	45	15	40	20	20	-10
ϕ_{ref} (deg)	-30	0	-20	-25	55	0	5	55	15	-10	-10	20

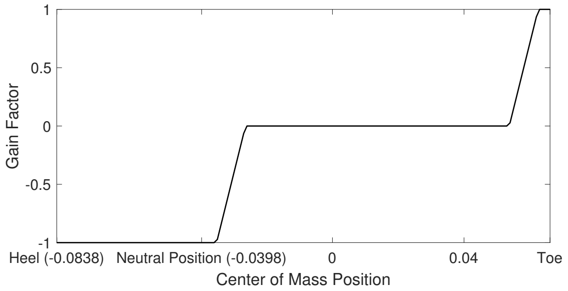

Figure S1. Plot of the multiplication factor defined by the dead zone parameters. The actual gain is the gain reported in table 2, multiplied with the factor. At the heel side, the sign is opposite to the toe, and the factor is -1, while at the toe it is equal to 1. Between 0 and the maximum, there is a gradual increase/decrease.

3 CORRELATION PLOTS FOR LENGTH AND FORCE FEEDBACK MODEL

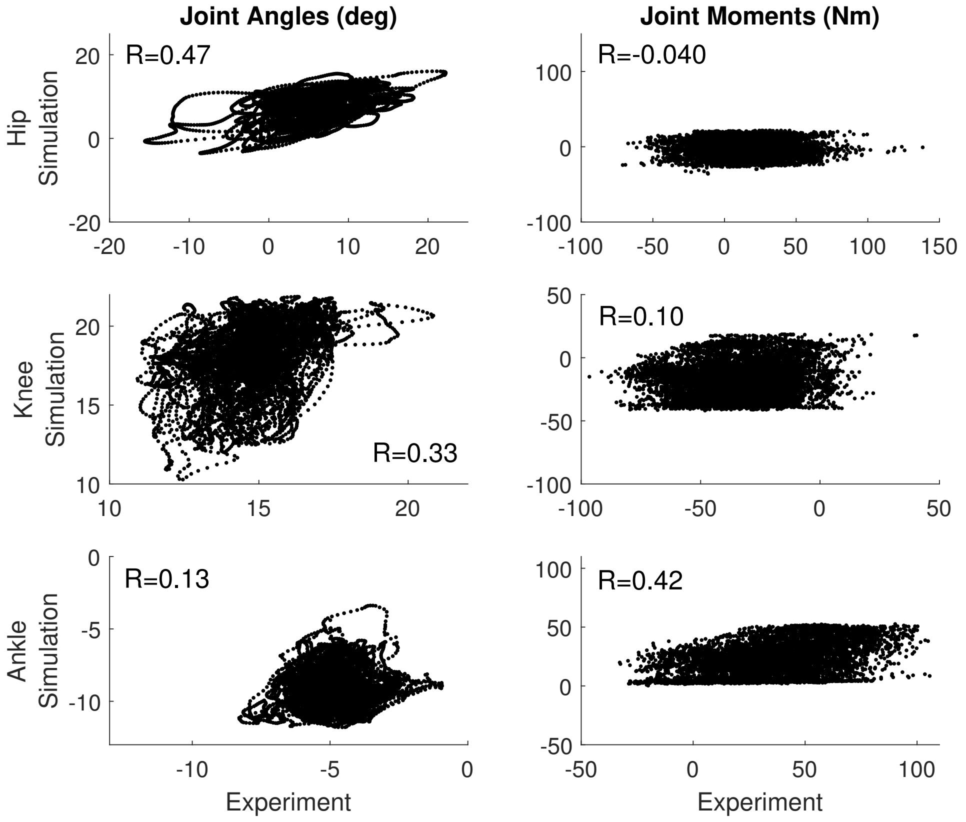

Figure S2. Correlation between the simulation with length feedback and the experiment for the joint angles (left) and joint moments (right) for all three joints.

4 RESULTS WITH DIFFERENT TIME DELAY

Figure S4 shows the time delay of the shows the joint angles and joint moments of the simulation with a shorter time delay versus the experiment. 10 ms was subtracted from all time delays as mentioned in table 2, to account for the time between stimulation and electromyography response in the experiments used to find the muscle time delay. The hip and ankle angle correlate moderately, with a higher correlation than for the base model (see table 4), while the correlation in the knee is weak and lower than in the base model. The joint moments correlated weakly, and negatively for the knee and hip. All correlations were significant.

Figure S5 shows the joint angles as a function of time for the simulation (red) and the experiment (black). The platform motion is also plotted for reference. The reference position of all joints is similar between the experiment and the simulation. The largest difference was three degrees for the ankle, while the difference was less than one degree for the hip and knee. Figure S6 shows the joint angles only for 60-80 seconds. The responses in all joints look very similar, especially in the ankle, while there is some more difference in the hip and knee.

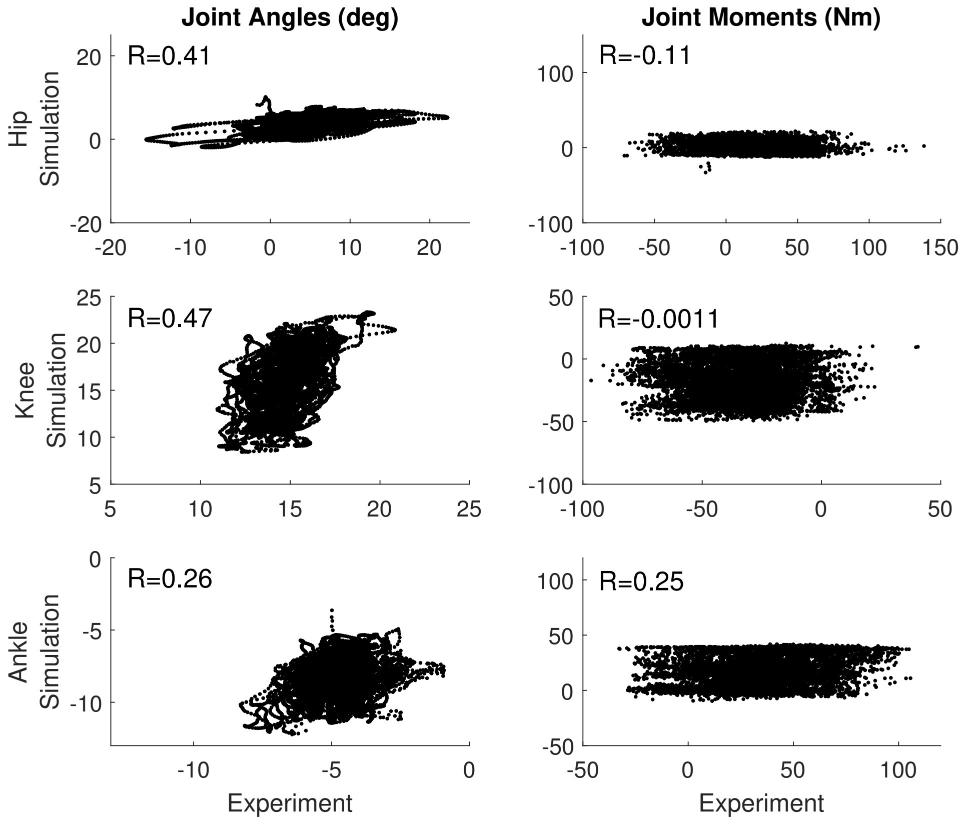

Figure S3. Correlation between the simulation with force feedback and the experiment for the joint angles (left) and joint moments (right) for all three joints.

Figure S7 shows the joint moments as a function of time for the simulation controlled with length and force feedback (red) and the experiment (black). The range of moments is smaller for the simulation than for the experiment, with smaller extremes in positive and negative direction.

5 RESULT WITH TIME DELAY IN THE CENTER OF MASS MEASUREMENT

Table S3 shows the controller parameters that were found for the base model, where a time delay was added in the measurement of the center of mass. Table S4 shows the correlations that were found with this controller. Both results are very similar to the controllers without a time delay in the center of mass.

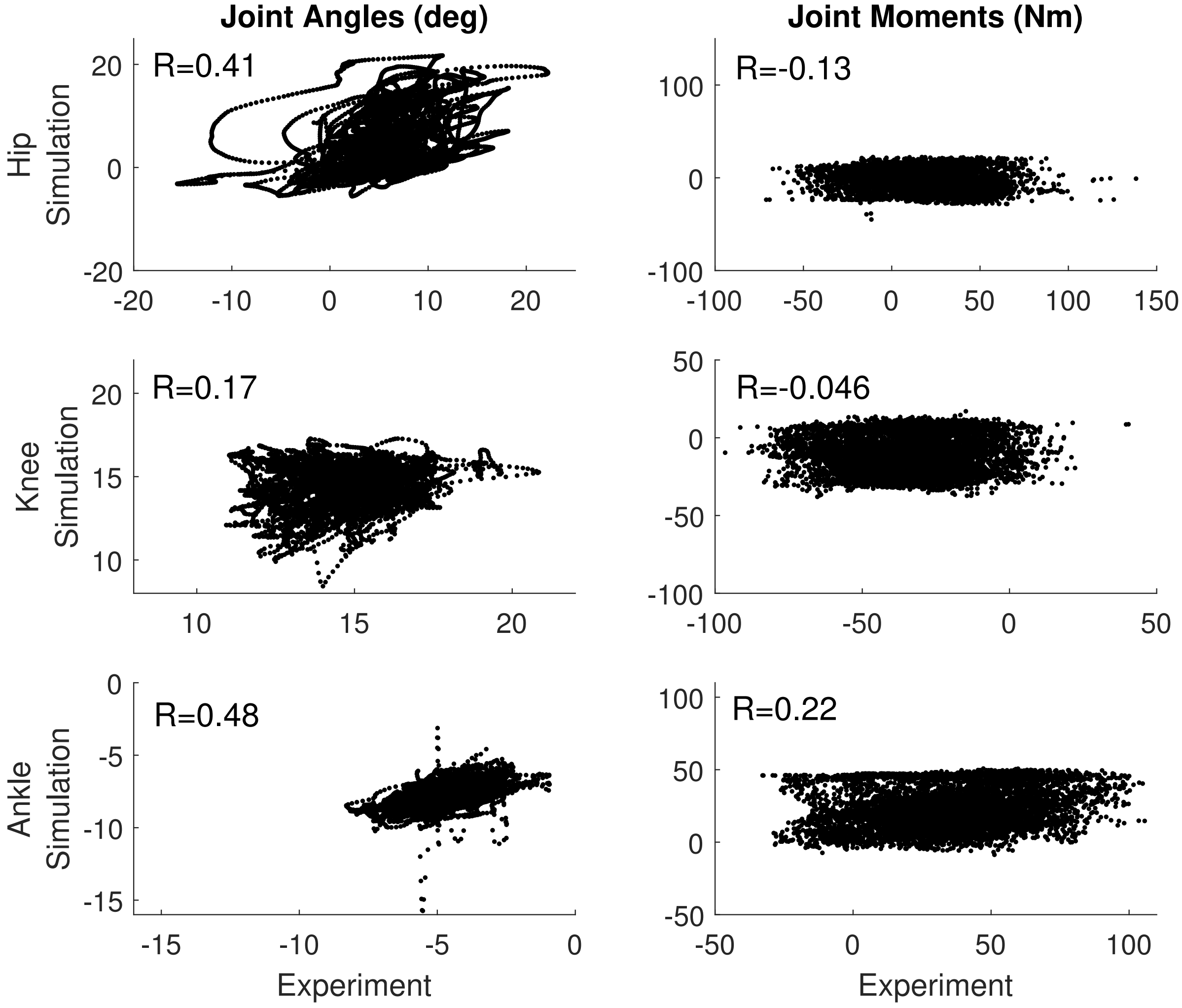

Figure S4. Correlation between the simulation controlled with the base model, but a shorter time delay, and the experiment for the joint angles (left) and joint moments (right) for all three joints.

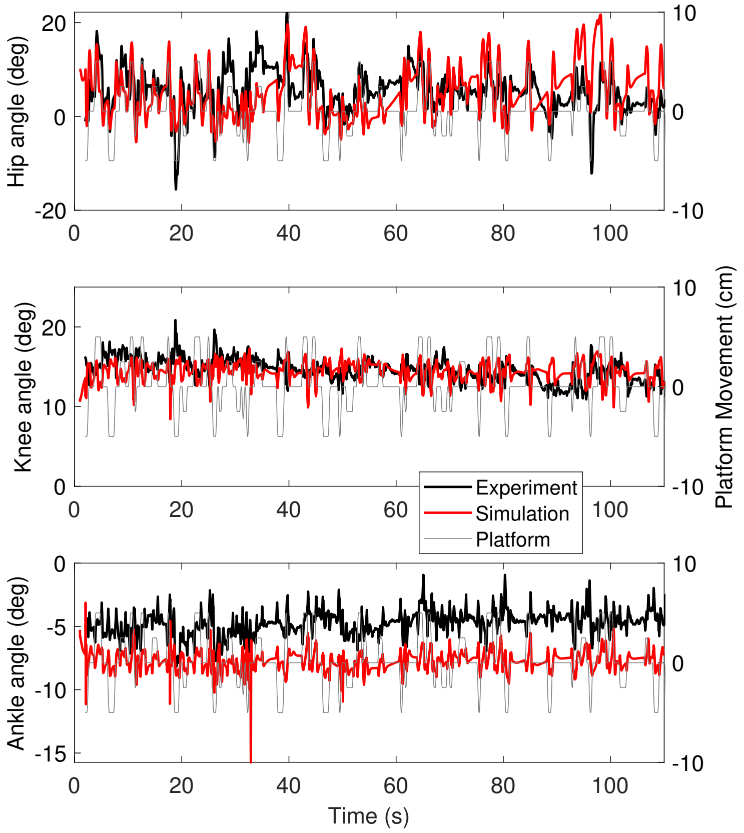

Figure S5. Joint angles as a function of time for the simulation controlled with different time delay (red) and experiment (black). The platform movement is shown in grey for reference.

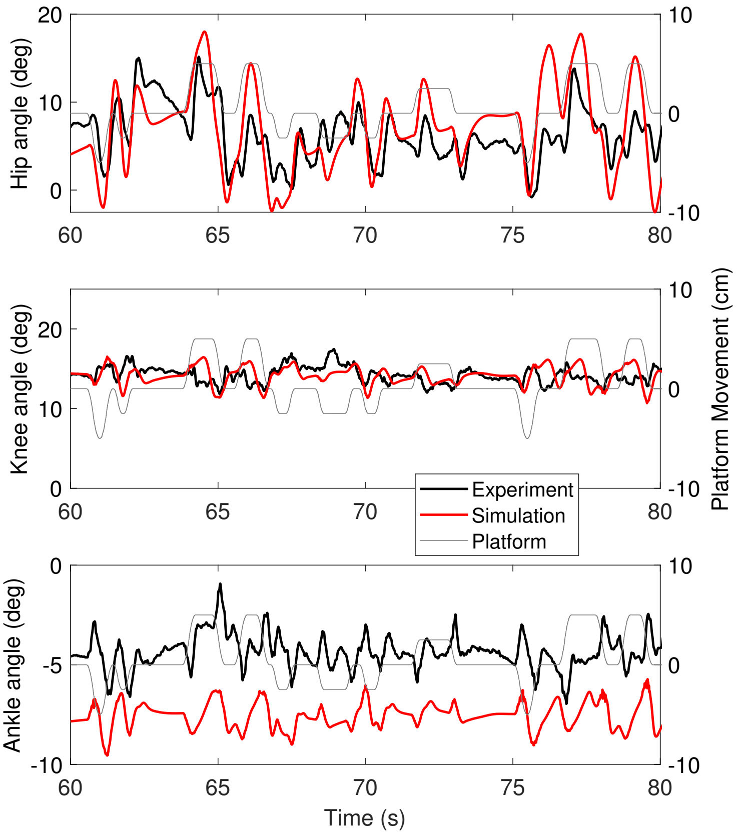

Figure S6. A zoom-in on the joint angles between 60 and 80 seconds for the simulation controlled with the model with different time delay (red) and experiment (black). The platform motion is shown in grey for reference.

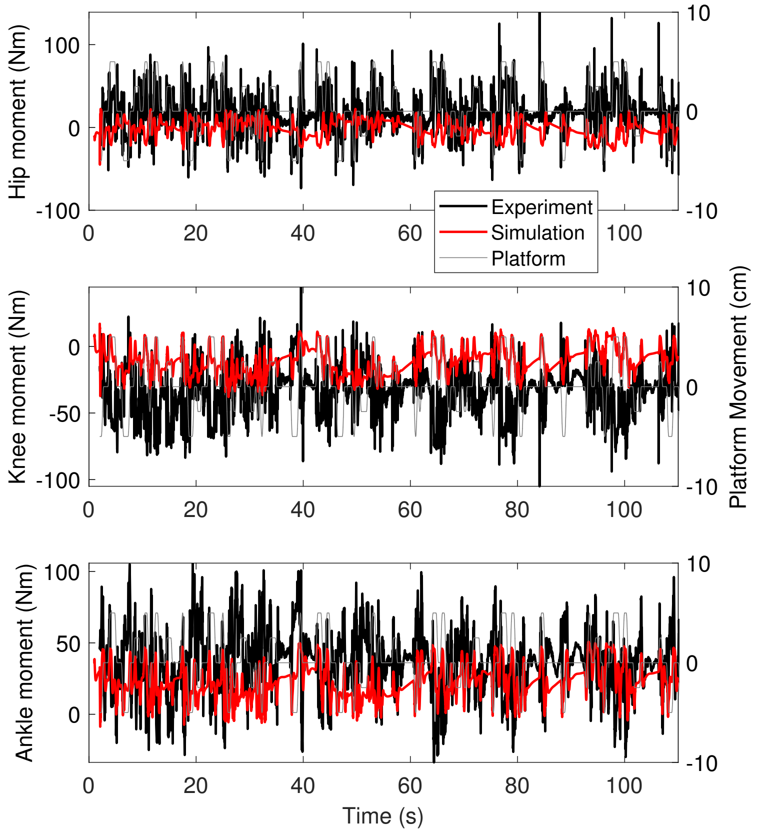

Figure S7. Joint moments as a function of time for the simulation controlled with different time delay (red) and experiment (black). The platform movement is shown in grey for reference.

Table S3. Optimized controller parameters for the controller with a time delay on the center of mass.

Parameters	Muscle	Base model, center of mass delay
z_{heel}		0.16
z_{toe}		0.53
x_{heel}		5.84 cm
x_{toe}		1.76 cm
u_0	Iliopsoas	0.20
	Gluteals	0.067
	Rectus Femoris	0.10
	Biceps Femoris LH	0.29
	Biceps Femoris SH	0.036
	Vastus	0.26
	Gastrocnemius	0.16
	Soleus	0.30
	Tibialis Anterior	0.095
G_{FFB}	Iliopsoas	0.64
	Gluteals	0.47
	Rectus Femoris	0.33
	Biceps Femoris LH	0.51
	Biceps Femoris SH	0.14
	Vastus	-0.090
	Gastrocnemius	0.95
	Soleus	1.45
	Tibialis Anterior	-0.75
G_{LFB}	Iliopsoas	0.98
	Gluteals	-0.46
	Rectus Femoris	-1.09
	Biceps Femoris LH	0.11
	Biceps Femoris SH	0.35
	Vastus	1.34
	Gastrocnemius	-1.07
	Soleus	0.98
	Tibialis Anterior	-0.44
$l_{CE(off)}$	Iliopsoas	1.01
	Gluteals	0.68
	Rectus Femoris	0.81
	Biceps Femoris LH	0.60
	Biceps Femoris SH	0.94
	Vastus	0.80
	Gastrocnemius	0.87
	Soleus	0.98
	Tibialis Anterior	0.74

Table S4. Correlation between simulated and experimental joint angles and joint moments controller with a time delay on the center of mass using the experimental data used in the optimization. A star indicates that the correlation is significant ($p \leq 0.0001$).

Control Model	Joint Angles			Joint Moments		
	Hip	Knee	Ankle	Hip	Knee	Ankle
Base Model	0.35*	0.37*	0.17*	0.0089	0.20*	0.41*
